# ST2 is reduced by high-dose omega-3 fatty acid treatment following acute MI and is correlated with reduction of the extracellular volume fraction of non-infarcted myocardium

**DOI:** 10.1186/1532-429X-18-S1-O130

**Published:** 2016-01-27

**Authors:** Bobby Heydari, Shuaib Abdullah, James V Pottala, Ravi V Shah, Siddique A Abbasi, Damien Mandry, Heidi Lumish, Udo Hoffmann, Evan Appelbaum, Jiazuo Feng, Ron Blankstein, Michael Steigner, Joseph P McConnell, William Harris, Michael Jerosch-Herold, Raymond Y Kwong

**Affiliations:** 1 Cardiology, University of Calgary, Calgary, AB Canada; 2 Cardiology, Brigham and Women's Hospital, Boston, MA USA; 3 Department of Internal Medicine, Sanford School of Medicine, Sioux Falls, SD USA; 4 Department of Medicine, Massachusetts General Hospital, Boston, MA USA; 5 Department of Radiology, Massachusetts General Hospital, Boston, MA USA; 6 Department of Medicine, Beth Israel Deaconess Medical Center, Boston, MA USA; 7 Health Diagnostic Laboratory, Richmond, VA USA

## Background

ST2, a member of the interleukin-1 receptor family, has been shown to be independently associated with myocardial strain and adverse cardiac events in patients with both ST-elevation and non-ST elevation myocardial infarction (MI). We sought to determine whether high-dose omega-3 fatty acid therapy (O-3FA) would reduce serum levels of ST2 following acute MI and whether ST2 levels correlated directly with measures of diffuse myocardial fibrosis within non-infarcted myocardium.

## Methods

We evaluated 358 patients who were enrolled in a randomized, double-blinded, placebo-controlled trial of high-dose O-3FA therapy post acute MI. All patients underwent 3T CMR (Tim Trio/Verio, Siemens, Germany) and evaluation of serum biomarkers at enrollment and after 6-months of randomized study therapy. Patients were followed for adverse cardiac events by study physicians at 6-month intervals thereafter.

## Results

**Figure 1:**
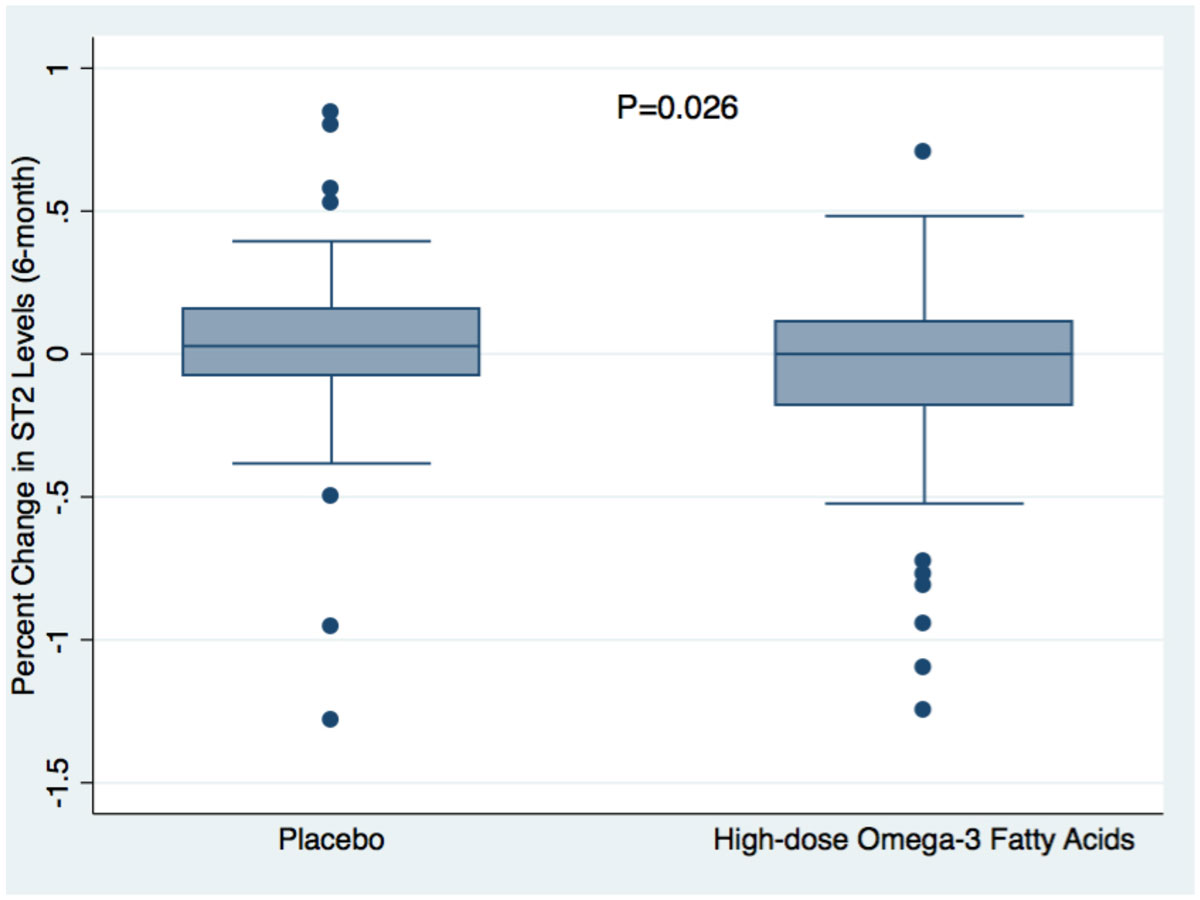
**Percent Change of Serum Biomarker ST2 Levels Post Treatment**. Percent change from baseline to post treatment (6 months) of ST2 levels for the high-dose omega-3 fatty acid treated group and placebo arm. P values are for comparisons of percent change in ST2 levels between the randomized treatment arms.

**Figure 2:**
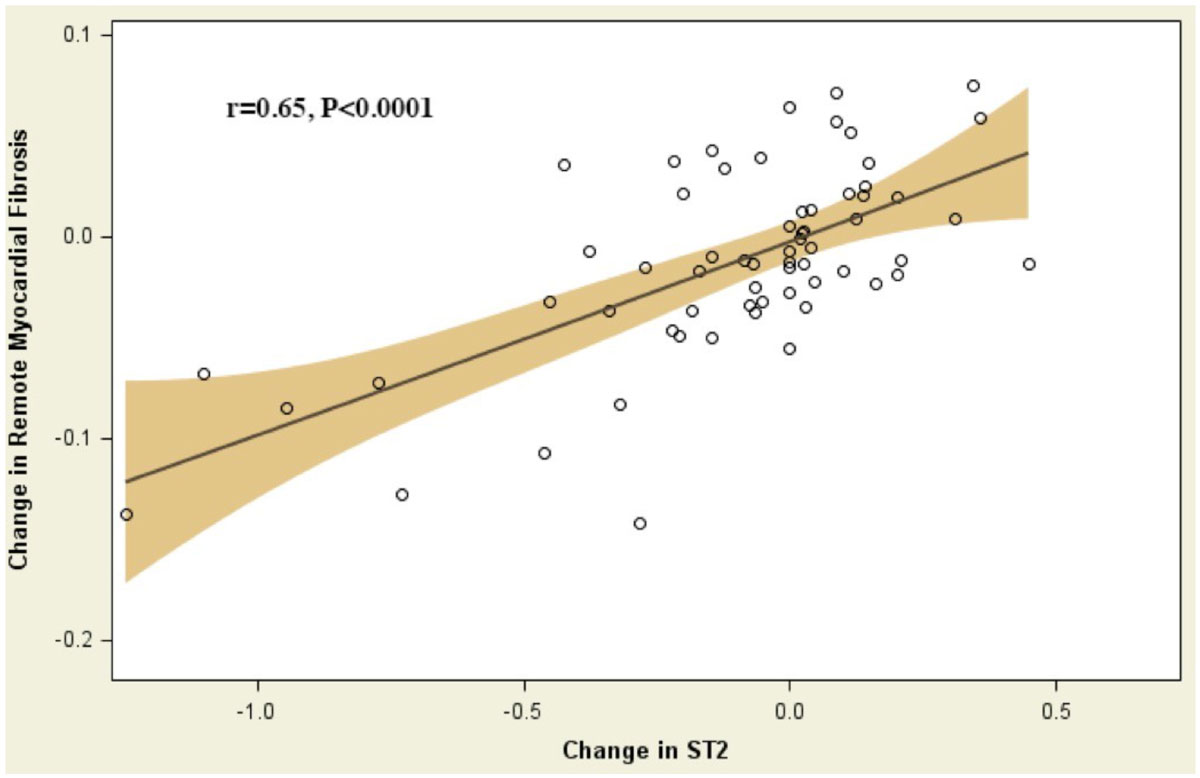
**Scatter Plot of Percent Change in Serum Biomarker ST2 versus Percent Change of Remote Myocardial Fibrosis Post Treatment**. Percent change from baseline to post treatment of the serum biomarker ST2 correlated against percent change in remote myocardial fibrosis by cardiac magnetic resonance imaging following 6 months of treatment with high-dose omega-3 fatty acids. P value is for Pearson correlation coefficient shown in figure.

Patients who received O-3FA treatment were more likely to have a history of coronary bypass surgery than placebo patients (p = 0.02), otherwise there were no baseline differences between treatment arms. ST2 levels were significantly reduced by O-3FA therapy as compared with placebo (Figure 1). By intention-to-treat analysis, O-3FA treatment was associated with a -7.9% reduction of ST2 (P = 0.03), and in adjusted analysis for covariates by -8.0% (P = 0.03). Amongst O-3FA treated patients, reduction of ST2 demonstrated a strong correlation with reduction of extracellular volume fraction within non-infarcted myocardium (r = 0.65, P < 0.0001, Figure 2). Baseline ST2 levels were the strongest unadjusted predictor (HR 5.2, 95% confidence interval 2.3-11.7, p < 0.0001) for all-cause mortality and congestive heart failure after a median of 2.3 years of follow-up.

## Conclusions

Serum ST2 level following acute MI is a strong prognostic marker of post-MI death and congestive heart failure. O-3FA treatment reduced ST2 levels, which were strongly correlated with reduction of the extracellular volume fraction within non-infarcted myocardium. ST2 may serve as a non-invasive serum biomarker of myocardial fibrosis, as well an independent predictor of adverse cardiac events following acute MI.

